# CONDUCTING CLINICAL TRIALS AMONG PERSONS WITH DEMENTIA: METHODS, CHALLENGES, AND OPPORTUNITIES FOR IMPROVEMENT

**DOI:** 10.1093/geroni/igz038.105

**Published:** 2019-11-08

**Authors:** Justine S Sefcik, Nancy Hodgson

**Affiliations:** 1 University of Pennsylvania School of Nursing, Philadelphia, Pennsylvania, United States; 2 University of Pennsylvania, Philadelphia, Pennsylvania, United States

## Abstract

Evidence-based interventions for persons with dementia and their caregivers are important to improve both their quality of life and quality of care. Conducting rigorous clinical trials is essential to build evidence, guide research, and inform clinical practice. Clinical trials involve multifaceted phases which present opportunities and challenges to the research team. In this presentation, we discuss obtaining consent, gathering information to inform refinement of an intervention, adaptation of an intervention, and fidelity checks to ensure the rigor of intervention delivery. The first session will discuss initial challenges faced when consenting nursing home residents with dementia and tailored approaches to enhance clinical trial enrollment. The second session will share highlights of usability testing of a novel mobile app to refine and improve its ease in a future clinical trial of use for persons with dementia and their caregivers. The third session will describe findings from focus groups that generated recommendations for culturally adapting an intervention to promote quality of life among older Latinos with dementia. The final session will discuss the advantages, disadvantages and ethical considerations of in-person fidelity monitoring during a home-based intervention clinical trial involving persons with dementia and their caregivers. Together, these presentations describe the complex nature of implementing clinical trials and highlight the important lessons learned to strengthen study implementation.

